# Prevalence of Obstructive Sleep Apnea in Patients With Diabetic Foot Ulcers

**DOI:** 10.3389/fendo.2020.00416

**Published:** 2020-07-14

**Authors:** Lihong Chen, Wanxia Ma, Weiwei Tang, Panpan Zha, Chun Wang, Dawei Chen, Fei Lei, Taomei Li, Xiangdong Tang, Xingwu Ran

**Affiliations:** ^1^Department of Endocrinology and Metabolism, Diabetic Foot Care Center, West China Hospital, Sichuan University, Chengdu, China; ^2^State Key Laboratory of Biotherapy, Sleep Medicine Center, Mental Health Center, Translational Neuroscience Center, West China Hospital, Sichuan University, Chengdu, China

**Keywords:** diabetic foot ulcer, obstructive sleep apnea, prevalence, polysomnography, wounds and injuries

## Abstract

**Objectives:** Diabetic foot ulcers (DFUs) are a considerable burden on patients and the healthcare service system. Patients with DFUs have many risk factors that might contribute to obstructive sleep apnea (OSA). The purposes of this study were to assess the prevalence of OSA and associated features in patients with DFUs.

**Methods:** Between July 2017 and June 2019, we recruited 245 consecutive patients who sought for treatment at West China Hospital because of DFUs. Polysomnography data from 127 Patients were included in the final analysis.

**Results:** Of the 127 patients, with a median age of 64 years (interquartile range, 55–73 years; range, 36–86 years) and a mean body mass index (BMI) 24.09 ± 0.37 kg/m^2^, 91 (72%) were men. The prevalence of OSA [apnea–hypopnea index (AHI) ≧5/h] was 92% in men and 97% in women (*P* = 0.304). Moderate to severe OSA (AHI ≧15/h) was noted in 44 men (48%) and 26 women (72%) (*P* = 0.015). The risk factors associated with the severity of OSA were sex, age, smoking, alcohol use, and duration of diabetes. After multivariable adjustment, duration of diabetes and age were independent predictive factors of the severity of OSA. No significant association was observed between BMI, waist circumference, Epworth score, and the severity of OSA. There were no significant associations between OSA and ischemic heart disease, cerebral infarction, hypertension, diabetic retinopathy, diabetic kidney disease, and peripheral artery disease.

**Conclusions:** The prevalence of OSA was high in patients with DFUs, with moderate to severe OSA accounting for more than half of the patients. Age and duration of diabetes were independent predictive factors of the severity of OSA.

## Introduction

Diabetic foot ulcers (DFUs) are one of the most serious complications of diabetic patients (1). Diabetic foot ulcers are common and are associated with a high risk of amputation. The 5-year survival is only 50–60% (2, 3). The cost is also huge. The estimated global cost of diabetes in 2015 was $1.3 trillion (4), and up to one-third of diabetes expenditure was on lower limb–related problems in the United States (5). Diabetic foot ulcers have become a very considerable burden on patients and the healthcare service system.

Obstructive sleep apnea (OSA) is characterized by episodic sleep state–dependent collapse of the upper airway, resulting in periodic reductions or cessations in ventilation, with consequent hypoxia, hypercapnia, or arousals from sleep (6). The prevalence of OSA is conservatively estimated to be 3% among women and 10% among men 30–49 years of age and 9% among women and 17% among men 50–70 years of age (7). Furthermore, patients with sleep-disordered breathing are more likely to have hypertension, stroke, heart failure, diabetes, car accidents, and depression (8, 9).

Multiple studies have shown a high prevalence of undiagnosed OSA in patients with type 2 diabetes mellitus (T2DM) (24–86%) (10–12). Furthermore, cohort studies have shown that the association of OSA and T2DM is bidirectional (12, 13). As a result, the International Diabetes Federation in 2008 recommended routine screening for OSA in patients with T2DM (14), and the Chinese Diabetes Society also recommended similar issues in Chinese guideline for the prevention and management of T2DM (2017 edition) (15).

There are more risk factors of OSA in patients with DFUs than diabetic patients without ulcers. In addition, diabetic-related foot disease was recognized as an independent risk factor of OSA (13). Therefore, it is very likely that patients with DFUs would have higher prevalence of OSA. Researchers have reported a higher prevalence of OSA in chronic wounds based on questionnaires (46%) (16). Furthermore, case reports and cohort studies have revealed that severe OSA might contribute to delayed wound healing (17, 18), and continuous positive airway pressure (CPAP) therapy might improve wound healing (17).

However, there is lack of data regarding the clinical, metabolic, outcomes, and economic impacts of OSA in patients with DFUs. Thus, it is necessary to examine the prevalence and characteristics of OSA in patients with DFUs. The primary aim of this study was to evaluate the prevalence and characteristics of OSA in DFUs.

## Methods

### Patients

We recruited 245 consecutive patients between July 2017 and June 2019 at West China Hospital when they sought for treatment because of DFUs. Because of the complexity and inconvenience of the polysomnography test, 118 patients (48%) did not accomplish the polysomnography test or acquire the sleep study data of high quality. No participant had previously been diagnosed sleep disorders or was being treated with CPAP. The Biomedical Research Ethics Committee of West China Hospital of Sichuan University approved the study. Because of the retrospective feature of the study, it was not necessary to obtain written informed consent from patients.

### Data Collection

The following demographics and clinical data were retrieved at the date of inclusion: age, sex, weight, height, duration of diabetes, history of smoking, and history of regular alcohol consumption. The medical document was examined to determine whether patients had diabetic complications including retinopathy, nephropathy, peripheral neuropathy, peripheral artery disease, and the presence of comorbidities such as hypertension, ischemic heart disease, and cerebrovascular diseases. We also included laboratory data including glycated hemoglobin (HbA_1c_), full blood count, serum lipids, and creatinine.

The standard overnight polysomnography was performed by certified technicians. They equipped patients with a portable, battery-powered polysomnography recorder (Somté; Compumedics, Abbotsford, Victoria, Australia) between 20:00 and 8:00 in the Department of Diabetic Foot Care Center of West China Hospital. Subjects were allowed to sleep based on their habitual sleep time. Derivations include electroencephalography (F4–M1, C4–M1, O2–M1, F3–M2, C3–M2, O1–M2), bilateral electro-oculography, electrocardiography, electromyography (submental and anterior tibialis), nasal pressure and thermal airflow, thoracoabdominal movements, and pulse oxygen saturation. Sleep was scored by a certified technician, blind to any diagnosis. Respiratory events were manually scored according to the 2012 American Academy of Sleep Medicine recommendations (19). Apnea was defined as a drop of at least 90% of airflow from baseline lasting 10 s or longer. Hypopnea was defined as a reduced airflow <30% of baseline for more than 10 s, in association with either a 3% oxygen desaturation or an arousal. We reported apnea–hypopnea index (AHI) by the average number of apnea and hypopnea events per hour of sleep. Obstructive sleep apnea was defined as usual clinical thresholds (normal, <5 events per hour; mild, 5–14.9 events per hour; moderate, 15–29.9 events per hour; and severe, ≥30 events per hour).

### Statistical Analysis

Statistical analysis was performed with Stata version 13 (Stata Corp). A *p* < 0.05 was considered statistically significant. We summarized data as either the number of patients (%), mean (SD), or median (interquartile range). We did the bivariate analysis with χ^2^-test, Student *t*-test, or Mann-Whitney *U*-test. We used logistic regression (partial proportional odds model) to assess the association between OSA severity and clinical variables and the association between risk factors and OSA severity. For the association analysis of OSA severity and risk of cardiovascular outcomes and diabetic complications, severity of OSA was assessed by AHI quartiles (Q1, 0–8.9 events per hour, Q2 9–17.5 events per hour, Q3 17.6–33.5 events per hour, Q4>33.5 events per h).

## Results

Two hundred forty-five consecutive patients were recruited between July 2017 and June 2019. Because 118 patients (48%) did not accomplish the polysomnography test or acquire the sleep study data of high quality, only 127 cases entered the final analysis.

Among the 118 patients who did not have sleep study data, 38 patients explicitly refused because they heard of the uncomfortable experience of the patients who accomplished the test in the same inpatient ward and did not want to have the same experience. Twenty-five patients ceased in the process of the test because they felt much uncomfortable and could not fall asleep. Twenty-three patients accomplished the test, but the quality of the result was too poor to report. Thirty-two medical records did not describe the reason why they refused the test. There were no significant differences in age, duration of diabetes, and diabetic comorbidities between patients included in the final study and those who were not included. There was significant difference in body mass index (BMI) and high-density lipoprotein cholesterol (LDL-c) (Supplementary File 1).

The demographic details of the 127 patients enrolled are displayed in Table 1. The median age was 64 years (55–73 years), with a mean BMI of 24.09 ± 0.37 kg/m^2^. The median duration of diabetes was 11 years, with a mean HbA_1c_ of 8.4 ± 0.2%. The median age was higher in women than in men (69 vs. 63, *P* = 0.03). There was significant difference in smoking, alcohol use, HbA_1c_, and LDL-c between men and women, whereas no significant difference in BMI, waist circumference, duration of diabetes, and total cholesterol was revealed between men and women. The prevalence of cardiovascular comorbidities and diabetic complications was relatively high in patients with DFUs (12–98%), with highest prevalence of diabetic peripheral neuropathy (98%) (Supplementary File 1). No significant difference between men and women was demonstrated in the prevalence of ischemic heart disease, cerebral infarction, hypertension, diabetic retinopathy, diabetic kidney disease, peripheral artery disease, and diabetic peripheral neuropathy.

**Table 1:** Demographic and clinical characteristics of patients.

	**Total (*n* = 127)**	**Men (*n* = 91)**	**Women (*n* = 36)**	***P***
Age (years)	64 (55–73)	63 (53–71)	69 (60–75)	0.034
<60	46 (36%)	37 (41%)	9 (25%)	—
≥60	81 (64%)	54 (59%)	27 (75%)	—
BMI (kg/m^2^)	24.09 ± 0.37	24.50 ± 0.47	23.17 ± 0.56	0.602
Waist circumference (cm)	90.72 ± 1.31	92.12 ± 1.67	87.61 ± 1.89	0.110
Smoking	79 (62%)	76 (84%)	3 (8%)	0.000
Alcohol use	62 (49%)	57 (63%)	5 (14%)	0.000
Epworth score	3 (0–8)	3 (0–7)	1.5 (0–8.5)	0.571
≥10	21 (17%)	15 (17%)	6 (16%)	0.602
Duration of diabetes (years)	11 (7–19)	11 (7–19)	11 (8–20)	0.671
HbA_1c_ (%)	8.38 ± 0.18	8.10 ± 0.20	9.07 ± 0.35	0.015
Cholesterol (mmol/L)	3.78 ± 0.10	3.64 ± 0.11	3.98 ± 0.19	0.119
Triglycerides (mmol/L)	1.64 ± 0.12	1.71 ± 0.15	1.46 ± 0.16	0.354
HDL-c (mmol/L)	1.07 ± 0.03	1.05 ± 0.04	1.14 ± 0.05	0.210
LDL-c (mmol/L)	1.97 ± 0.08	1.86 ± 0.09	2.25 ± 0.15	0.023

*Data are presented as number of participants (%), mean (SD), or median (interquartile range). BMI, body mass index; HbA_1c_, hemoglobin A_1c_; HDL-c, high-density lipoprotein cholesterol; LDL-c, low-density lipoprotein cholesterol. P-values are indicated for the comparison between men and women*.

**Figure 1:**
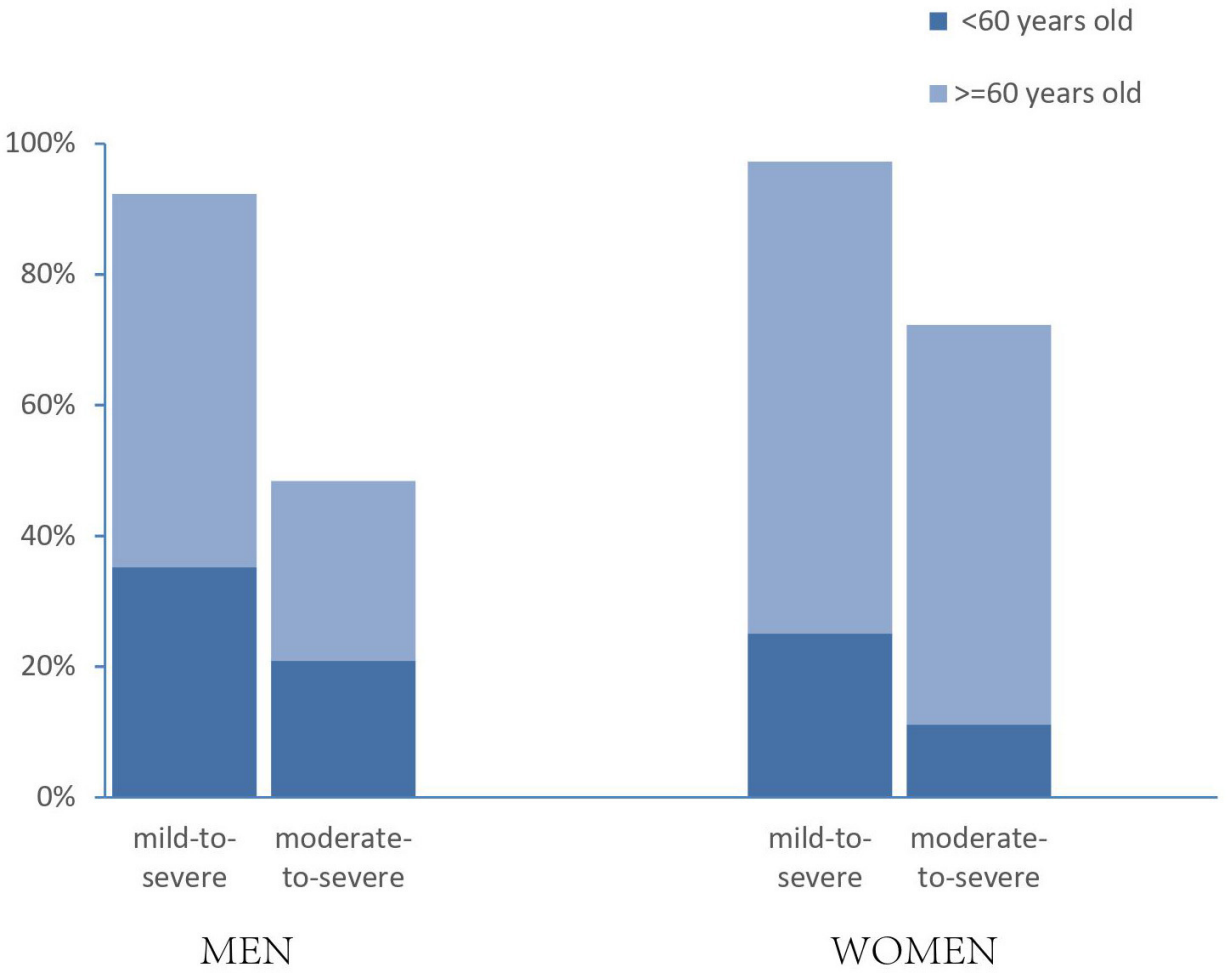
Prevalence estimates of OSA in patients with diabetic foot ulcer, by gender and age. “Mild to severe” OSA was defined as an AHI of 5 or more events per hour; “moderate to severe” was defined as an AHI of 15 or more events per hour. *P* = 0.015 between men and women in moderate to severe OSA; OSA differed by age in moderate-to-severe women (*P* = 0.032).

We identified 84 men (92%) and 35 women (97%) with OSA (*P* = 0.304), whereas moderate to severe OSA was noted in 44 men (48%) and 26 women (72%) (*P* = 0.015). To assess the effect of age on prevalence of OSA, we divided patients into two categories, younger than 60 and 60 years or older. There was a significant difference in moderate to severe OSA between men and women in the older patients [odds ratio (OR) = 0.20, 95% confidence interval (CI) = 0.65–0.59, *P* = 0.004], whereas no difference was revealed in the younger group (OR = 1.32, 95% CI = 0.31–5.71, *P* = 0.710). In addition, Moderate to severe OSA was much higher in the group of older women compared with those in the younger group (22/27 vs. 4/9, *P* = 0.032), whereas this phenomenon was not revealed in men (*P* = 0.636) (Figure 1).

The main risk factors associated with the severity of OSA were sex, age, smoking, alcohol use, and duration of diabetes. However, no association was observed between severity of OSA and BMI, waist circumference, and presence of daytime sleepiness, measured with the Epworth Sleepiness Scale (Table 2). A multivariable logistic regression (using partial proportional odds model) was used to examine which of these risk factors might be independent risk factors for OSA. Duration of diabetes and age were the independent predictive factors of severity of OSA (Table 3).

**Table 2:** Risk of OSA according to related factors, by severity.

	**Mild to severe**		**Moderate to severe**		**Severe**	
	**OR (95% CI)**	***P***	**OR (95% CI)**	***P***	**OR (95% CI)**	***P***
Age (per 10-year increment)	1.24 (0.66–2.32)	0.500	1.21 (0.88–1.66)	0.232	1.49 (1.05–2.09)	0.023
Sex	0.34 (0.04–2.89)	0.325	0.36 (0.16–0.83)	0.017	0.56 (0.25–1.25)	0.157
BMI (kg/m^2^)						
24–28 (vs. <24)	0.76 (0.18–3.22)	0.712	1.50 (0.70–3.18)	0.290	1.71 (0.77–3.81)	0.19
>28 (vs. <24)	—	—	1.34 (0.69–2.61)	0.380	1.24 (0.63–2.44)	0.53
Smoking	0.22 (0.03–1.88)	0.168	0.37 (0.17–0.80)	0.012	0.42 (0.19–0.90)	0.026
Alcohol use (yes vs. no)	0.55 (0.13–2.41)	0.429	0.45 (0.22–0.92)	0.029	0.48 (0.22–1.03)	0.059
Epworth score (≥10 vs. <10)	1.41 (0.16–12.1)	0.752	1.10 (0.43–2.84)	0.838	0.81 (0.29–2.27)	0.691
Duration of diabetes (per 5-year increment)	2.15 (1.25–3.67)	0.005	1.48 (1.11–1.97)	0.007	1.15 (0.85–1.55)	0.363
Waist circumference						
Q2 (vs. Q1)	1.06 (0.06–18.4)	0.967	1.1 (0.28–4.25)	0.890	0.91 (0.23–3.52)	0.890
Q3 (vs. Q1)	1.03 (0.25–4.30)	0.967	1.18 (0.59–2.35)	0.632	0.74 (0.36–1.50)	0.406
Q4 (vs. Q1)	0.84 (0.37–1.93)	0.682	0.92 (0.60–1.41)	0.691	0.96 (0.62–1.48)	0.847

“*Mild to severe” OSA was defined as an AHI of 5 or more events per hour; “moderate to severe” was defined as an AHI of 15 or more events per hour; “severe” was defined as an AHI of 30 or more events per hour. The cutoff points for waist circumference were based on quartiles; Q1, <84 cm; Q2, 84–89 cm; Q3, 90–97 cm; Q4, >97 cm. BMI, body mass index; OR, odds ratio; CI, confidence interval*.

**Table 3:** Multivariable logistic regression of risk factors of the severity of OSA.

	**Mild to severe**		**Moderate to severe**		**Severe**	
	**OR (95% CI)**	***P***	**OR (95% CI)**	***P***	**OR (95% CI)**	***P***
Age (per 10-year increment)	1.670 (0.475–5.866)	0.424	0.792 (0.435–1.439)	0.443	2.096 (1.069–4.111)	0.031
Sex	2.467 (0.0432–140.8)	0.662	3.056 (0.492–18.97)	0.230	2.788 (0.568–13.68)	0.206
Smoking	0.380 (00754–19.18)	0.629	0.235 (0.0411–1.339)	0.103	0.359 (0.0842–1.535)	0.167
Alcohol use (yes vs. no)	0.422 (0.0691–2.573)	0.349	0.504 (0.188–1.354)	0.174	0.627 (0.228–1.725)	0.366
Duration of diabetes (per 5-year increment)	2.807 (1.142–6.899)	0.025	2.186 (1.302–3.671)	0.003	0.721 (0.376–1.384)	0.326
BMI (kg/m^2^)	0.836 (0.344–2.032)	0.693	1.014 (0.737–1.394)	0.932	1.327 (0.961–1.833)	0.086
Waist circumference (cm)	1.030 (0.805–1.317)	0.817	0.970 (0.897–1.049)	0.443	0.932 (0.847–1.026)	0.153

“*Mild to severe” OSA was defined as an AHI of 5 or more events per hour; “moderate to severe” was defined as an AHI of 15 or more events per hour; “severe” was an AHI of 30 or more events per hour. BMI, body mass index; OR, odds ratio; CI, confidence interval*.

**Figure 2:**
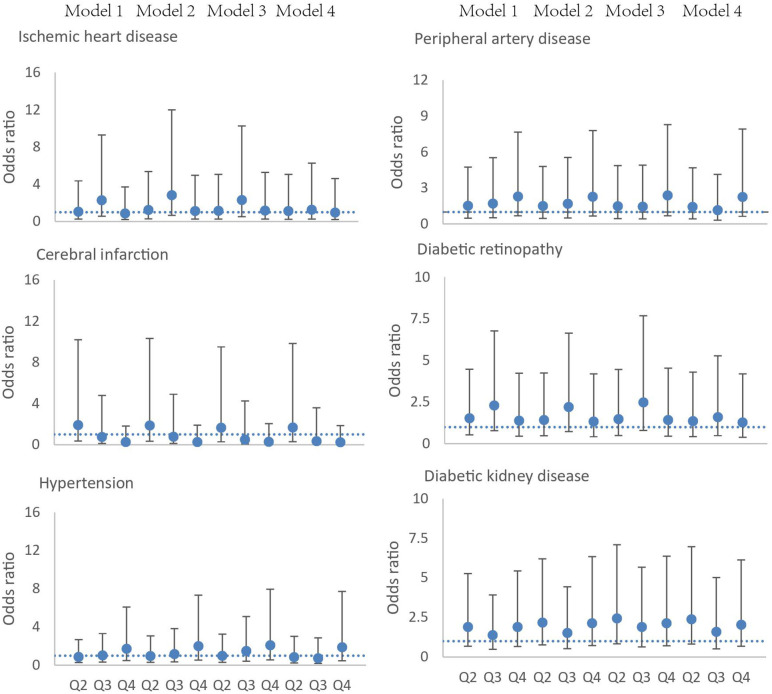
Association of the severity of OSA and ischemic heart disease, cerebral infarction, hypertension, peripheral artery disease, diabetic retinopathy, and diabetic kidney disease. Circles represent the odds ratio, and bars represent the 95% confidence interval. The severity of OSA is defined according to apnea–hypopnea index quartiles (Q1, 0–8.9 events per hour, Q2 9–17.5 events per hour, Q3 17.6–33.5 events per hour, Q4 >33.5 events per h). Model 1, adjusted for age and sex; model 2, adjusted for age, sex, smoking, and alcohol usage; model 3, adjusted for age, sex, smoking, alcohol usage, and body mass index; model 4, age, sex, smoking, alcohol usage, body mass index, and duration of diabetes.

In order to assess the associations between the severity of OSA and cardiovascular outcomes and diabetic complications, we divided the severity of OSA by AHI quartiles and adjusted logistic models to account for potential confounding risk factors. Four models were employed to evaluate the associations. There were no significant associations between OSA and ischemic heart disease, cerebral infarction, hypertension, diabetic retinopathy, diabetic kidney disease, and peripheral artery disease throughout all four models (Figure 2). The association between OSA and diabetic peripheral neuropathy cannot be calculated, because there was a rather high prevalence of diabetic peripheral neuropathy in patients with DFUs (98%) (Supplementary File 2).

## Discussion

The OSA prevalence in our study was rather high, with more than 90% of patients with DFUs experiencing at least mild OSA and more than half having at least moderate OSA. The population-based Swiss HypnoLaus study using the most recent polysomnographic recording techniques revealed a high prevalence of 72% for mild to severe OSA in general population older than 40 years, with a prevalence of moderate to severe at 23.4% in women and 49.7% in men (20). Many studies have shown a high prevalence of undiagnosed OSA in patients with T2DM (24–86%) (10–12). Furthermore, cohort studies have shown that the association of OSA and T2DM is bidirectional (12, 13). In a study from the United States of 249 patients with chronic wounds, the prevalence of OSA was 46%, higher than that in the general middle-aged population. However, only 50 patients underwent an in-home sleep study (16). In a study from the United Kingdom of 94 patients with DFUs, the researchers found that 64% of patients had a STOP-BANG score ≥4. However, the questionnaire is not a recommended tool for diagnosing OSA in adults in the absence of polysomnography or home sleep apnea testing (21). The prevalence of OSA is highly dependent on technical factors. Polysomnography is recommended by the American Academy of Sleep Medicine as the gold standard test to diagnose OSA. The high prevalence of risk factors of OSA, such as aging and heart failure, might contribute to the high prevalence of OSA in DFUs. However, it could be because that we used a more modern method of measuring OSA, polysomnography.

In our study, we found that female patients with DFUs had a higher proportion of moderate to severe OSA than male patients. This was interesting because, in previous studies, the male sex was recognized as a risk factor of OSA (22). When we divided the patients into two categories (younger than 60 years vs. older than 60 years), no difference was revealed in the younger group, whereas in the older group, female patients had a higher proportion of moderate to severe OSA. Studies have demonstrated that hormonal status affects the prevalence of OSA (20, 23). Lower progesterone stimulation of upper-airway muscles and ventilation may contribute to the higher OSA among older women because of the increased proportion of postmenopausal women (24). Menopause, independent of age and BMI, is a risk factor of OSA (9). In addition, the median age was higher in women than in men in our study; this might contribute to the high proportion of moderate to severe OSA in women. This is because age is another important risk factor of OSA (25, 26).

In our study, sex, age, smoking, alcohol use, and duration of diabetes were recognized as risk factors of OSA. However, multivariate logistic regression analysis demonstrated that age and duration of diabetes were the independent predictive factors of severity of OSA. This finding is consistent with the results of a study investigating the prevalence of OSA in hospitalized patients with T2DM in China (11). It seems that increasing age has more positive association with severe OSA in women rather than in men. More than 60% of patients in our study were older than 60 years. Cardiovascular events, such as heart failure and ischemic heart disease, increased dramatically with longer duration of diabetes among older patients (27, 28). Heart failure and ischemic heart disease are both risk factors of OSA. Thus, it is reasonable to consider the duration of diabetes as contributing factor to the severity of OSA.

We do not find associations between the severity of OSA and ischemic heart disease, cerebral infarction, hypertension, diabetic retinopathy, diabetic kidney disease, and peripheral artery disease. Previous studies have demonstrated association between severity of OSA and diabetes, hypertension, depression, myocardial infarction, stroke, and road traffic accidents (8, 9, 20). Studies in patients with diabetes also show association between severity of OSA and diabetic peripheral neuropathy and diabetic retinopathy (29–31). However, in our study, the relatively high prevalence of diabetic peripheral neuropathy in patients with DFU has made it impossible to predict the effect of OSA. Furthermore, diabetes is a major contributing factor of cardiovascular events and diabetic complications; the higher prevalence of cardiovascular events in patients with DFUs might mask the importance of OSA on the prevalence of these conditions.

The strengths of our study are that it was one of the first studies that investigated the prevalence of OSA in patients with DFUs. Moreover, polysomnographic recordings and modern scoring criteria were applied to ensure the validity of results in view of current recommendations (21). The main limitations were that 48% of patients did not have the sleep study data and were not included in final analysis, which might have caused selection bias. In comparison of the demographic characteristics between patients included and those not included, there were no significant differences in age and duration of diabetes, which were the risk factors of sleep apnea. Although there was significant difference in BMI, the difference of mean was “1.2 kg/m^2^.” We do not think it is big enough to have clinical significance to influence the results (Supplementary File 1). It is reasonable to consider whether the presence of symptoms influenced the decision of the patients to accomplish the sleep study. In clinical practice, we asked all patients to be screened for sleep apnea using polysomnography, rather than testing patients according to whether they had symptoms. In addition, the median Epworth score was 3 (0–8), and 83% of patients in this study had an ESS score of <10. This was similar with that in Heinzer and colleagues' (20) study in the general population. Thus, we do not think whether the presence of symptoms influenced the decision to accomplish the sleep study. Furthermore, because our study was similar in terms of age, sex, BMI, ischemic heart disease, cerebral infarction, peripheral artery disease, diabetic kidney disease, and diabetic retinopathy to a large cohort of DFU patients in China (32), therefore, we believe that our results are probably very close to the prevalence of patients with DFUs. Second, the relatively small sample number might also contribute to bias. Further studies with larger sample sizes are warranted to confirm the high prevalence of sleep apnea.

## Conclusion

The prevalence of OSA in patients with DFUs was considerably high, with moderate to severe OSA accounting for more than half of the patients. Age and duration of diabetes were independent predictive factors of severity of OSA. When considered alongside the effect of OSA on hypertension, myocardial infarction, stroke, depression, wound healing, and road traffic accidents reported in previous studies, it is necessary to conduct further research to verify the impact of OSA on patients with DFUs, including wound healing.

## Data Availability Statement

All datasets generated for this study are included in the article/Supplementary Material.

## Ethics Statement

The studies involving human participants were reviewed and approved by Biomedical Research Ethics Committee of West China Hospital of Sichuan University. Written informed consent for participation was not required for this study in accordance with the national legislation and the institutional requirements.

## Author Contributions

LC: study design, hypothesis conception, statistical analysis, and manuscript writing. WM: study design, data collection, data analysis, and manuscript revision. WT, PZ, CW, and DC: study design and data collection. FL, TL, and XT: data collection and data analysis. XR: hypothesis conception, study design, and manuscript revision. All authors: contributed to the article and approved the submitted version.

## Conflict of Interest

The authors declare that the research was conducted in the absence of any commercial or financial relationships that could be construed as a potential conflict of interest.
