# Supplementary material for: Prevalence of Obstructive Sleep Apnea in Patients With Diabetic Foot Ulcers
Source: Front Endocrinol (Lausanne). 2020 Jul 14;11:416. doi: 10.3389/fendo.2020.00416 (PMC7371781; doi:10.3389/fendo.2020.00416)
Supplement: Supplementary file 1 [file Data_Sheet_1.pdf]

Supplementary File 1. Comparison of demographic and clinical characteristics between patients who were included in final analysis and those who were not included

	Total (n=245)	Included (127)	Not included (118)	P value
age (years)	64±12	63±12	66±12	0.129
＜60	82(33%)	46(36%)	36(31%)	-
⩾60	163(67%)	81(64%)	82(69%)	-
BMI (Kg/m^2)	23.5±3.21	23.96±3.03	22.71±3.35	0.008
Waist circumstance (cm)	90.08±10.75	90.72±11.24	88.96±9.87	0.396
Smoking	125(59%)	79(63%)	46(53%)	0.152
Alcohol use	96(45%)	62(49%)	34(39%)	0.144
Duration of Diabetes (years)	10(6-18)	11(7-19)	10(5-15)	0.084
HbA1c (%)	8.3±2.1	8.38±2.00	8.31±2.19	0.779
Cholesterol (mmol/l)	3.78±1.10	3.74±1.11	3.81±1.09	0.614
Triglycerides (mmol/l)	1.62±1.15	1.64±1.34	1.60±0.90	0.814
HDL-c (mmol/l)	1.20±0.56	1.07±0.38	1.35±0.68	0.000
LDL-c (mmol/l)	1.93±0.90	1.97±0.87	1.88±0.93	0.406
Ischemic heart disease	57(23%)	29(23%)	28(24%)	0.895
Peripheral artery disease	143(59%)	79(70%)	64(56%)	0.301
Hypertension	169(69%)	89(71%)	80(68%)	0.631
Diabetic retinopathy	102(42%)	48(38%)	54(46%)	0.225
Diabetic peripheral neuropathy	239(98%)	123(98%)	116(98%)	0.705
Diabetic kidney disease	128(52%)	61(48%)	67(57%)	0.191

Data are presented as number of participants (%), or mean (SD), or median (IQR). BMI, body mass index; HbA1c, hemoglobin A1c; HDL-c, high-density lipoprotein cholesterol; LDL-c, low-density lipoprotein cholesterol.

Supplementary File 2. Comorbidities and complications of patients with diabetic foot ulcers

	Total (n=127)	Men (n=91)	Women (n=36)	p value
Ischemic heart disease	30(24%)	23(26%)	6(17%)	0.284
Cerebral infarction	15(12%)	10(11%)	5(14%)	0.648
Heart failure	61(48%)	91(48%)	17(47%)	0.909
Peripheral artery disease	69(56%)	49(55%)	20(57%)	0.833
Hypertension	89(71%)	64(71%)	25(69%)	0.853
Diabetic retinopathy	48(38%)	36(40%)	12(33%)	0.486
Diabetic Peripheral neuropathy	123(98%)	88(98%)	35(97%)	0.853
Diabetic kidney disease	61(48%)	45(50%)	16(44%)	0.573

Supplementary File 3. Comparison of daytime sleepiness between this study and Heinzer's study

		Total	Men	Women
This study	Epworth score	3(0-8)	3(0-7)	2(0-8)
	>10	17%	17%	17%
Heinzer's study	Epworth score	6(3-9)	6(4-9)	5(3-8)
	>10	12%	14%	10%

Data are presented as number of participants (%), or median (IQR).
